# Analysis of Airborne *Betula* Pollen in Finland; a 31-Year Perspective

**DOI:** 10.3390/ijerph6061706

**Published:** 2009-05-26

**Authors:** Eija Yli-Panula, Desta Bey Fekedulegn, Brett James Green, Hanna Ranta

**Affiliations:** 1 Department of Teacher Education, University of Turku, Finland; 2 Aerobiology Unit, University of Turku, Finland; E-Mail: Hanna.Ranta@utu.fi; 3 Biostatistics and Epidemiology Branch, Health Effects Laboratory Division, National Institute for Occupational Safety and Health, Centers for Disease Control and Prevention, Morgantown, West Virginia, USA; E-Mail: djf7@cdc.gov; 4 Allergy and Clinical Immunology Branch, Health Effects Laboratory Division, National Institute for Occupational Safety and Health, Centers for Disease Control and Prevention, Morgantown, West Virginia, USA; E-Mail: dox6@cdc.gov

**Keywords:** allergen, *Betula* spp., birch, biometeorology, temperature

## Abstract

In this 31-year retrospective study, we examined the influence of meteorology on airborne *Betula* spp. (birch) pollen concentrations in Turku, Finland. The seasonal incidence of airborne birch pollen in Turku occurred over a brief period each year during spring (April 30 – May 31). Mean peak concentrations were restricted to May (May 5 to 13). Statistically significant increases in the annual accumulated birch pollen sum and daily maximum values were observed over the study period. Birch pollen counts collected in April were retrospectively shown to increase over the duration of the study. Increases in April temperature values were also significantly associated with the earlier onset of the birch pollen season. Furthermore, the number of days where daily birch pollen concentrations exceeded 10 and 1,000 grains/m^3^ also increased throughout the study period. These data demonstrate that increases in temperature, especially during months preceding the onset of the birch pollen season, favor preseason phenological development and pollen dispersal. Birch pollen derived from other geographical locations may also contribute to the aerospora of Turku, Finland. To date, the public health burden associated with personal exposure to elevated birch pollen loads remains unclear and is the focus of future epidemiological research.

## 1. Introduction

Bioaerosols emanating from botanical sources pose a significant environmental health risk. Airborne *Betula* spp. (birch) pollen is one of the most recognized aeroallergens in northern European countries. Personal exposure to as few as 10 grains/m^3^ is known to exacerbate seasonal allergic rhinitis and asthma in birch sensitized individuals [1–3]. In Scandinavia, approximately 10–20 % of the atopic population is sensitized to pollen aeroallergen sources [1,4–6]. During the last two decades allergic sensitization has increased throughout the developed world [3,7]. Exposure to elevated concentrations of birch pollen has been proposed to be a potential etiological agent associated with this increase. However, variables that influence birch pollen dispersal such as the long-term effects of meteorology on flower phenology and inflorescence development remain uncharacterized in many Scandinavian environments.

Birch pollen is one of the most abundant aeroallergens in Europe. The birch inflorescence is characterized by flowers that produce millions of pollen grains that disseminate into the atmosphere following wind disturbance. Concentrations of birch pollen, as with most other anemophilous tree pollen types, may fluctuate year to year by more than an order of magnitude [8–10]. In boreal climates, the annual variation in airborne pollen counts may be even more extreme [11]. Temperature is an important predictor of the timing of birch flower phenology in boreal regions [12,13]. Meteorological parameters especially spring temperature, break dormancy and facilitate the development of inflorescences and pollen [14]. Recent studies have hypothesized that elevated temperature during this period permits the preseason development of inflorescences and enables early pollen dispersal [15,16]. This phenomenon is thought to extend and intensify birch pollen seasons across many European regions. To date, the long-term influence of meteorological variables immediately preceding the birch pollen season requires further longitudinal assessment in Turku, Finland.

In this retrospective study, we aimed to understand the impacts of variability in meteorological parameters on the dispersal of birch pollen in a Finnish environment. This information will assist in planning allergen avoidance strategies for birch sensitized individuals as well as help to provide unique insight into the increasing prevalence and future burden of allergic diseases in Finland and other Scandinavian countries.

## 2. Materials and Methods

### 2.1. Study Area

Finland is classified as a temperate coniferous-mixed forest zone. The city of Turku is located on the south western coastal plain of Finland (60° 27′ N 22° 17′ E) and close to the Baltic Sea. The pollen trapping site was situated at Turku University, approximately one kilometer north east of the city center and eight kilometers south west of the coast. The elevation of the trapping site was 59 meters above sea level and was located on top of a Turku University building. The site was predominantly surrounded by residential premises occupied by various plant species including; *Acer platanoides*, *Alnus* spp., *Artemisia* spp., *Betula pendula*, *B. pubescens*, *Picea abies*, *Pinus sylvestris*, *Populus tremula*, *Sorbus* spp., *Tilia cordata*, and Poaceae species.

### 2.2. Pollen Sampling Methods

Birch pollen sampling occurred in Turku, Finland, from 1974 to 2004 (data kindly provided by The Finnish Aerobiology Unit). Airborne birch pollen was collected using a seven-day Burkard volumetric sampler (Burkard Manufacturing Co. Ltd., Rickmansworth, Hertfordshire, UK). Air sampling methods followed the aerobiological standards set by the British Aerobiology Federation [17]. The Burkard sampler was calibrated to continuously sample air at ten liters per minute and the atmospheric particulate matter was deposited onto tapes coated with a thin film of mounting medium. Pollen grains were resolved using light microscopy, stained with Calberla's staining solution, and quantified using randomized fields according to the methods of Mäkinen [18]. Birch pollen counts were converted to daily averages per cubic meter of air. Due to similar morphological characteristics, the two *Betula* species, *B. pendula* (silver birch) and *B. pubescens* (downy birch), were included in the same *Betula* pollen category [10].

### 2.3. Meteorological Data

The meteorological data was obtained from the Finnish Institute of Meteorology. The Turku research station is situated at Turku airport, approximately 20 kilometers from the trapping site. The data included average temperature and precipitation. The annual mean temperature is defined as the mean of daily average temperature for the study period (31 years) and is also referred to as the long-term average temperature. The yearly total precipitation was defined as the sum of monthly total precipitation for the specified year.

### 2.4. Statistical Analyses

Data corresponding to 31 years (1974–2004) of pollen monitoring and meteorological records were documented graphically and evaluated statistically. Daily pollen counts were classified into three categories (≥10, ≥100, ≥1,000 grains/m^3^), a standard that was developed by The Aerobiology Unit, University of Turku, Finland [2]. Regression analyses and smoothing were used to detect long-term trends in daily average precipitation, temperature, and pollen concentrations in the air. The moving average was calculated to provide an objective measure of trend direction by smoothing the meteorological and birch pollen data [19]. This was accomplished by using the method of moving averages; where moving averages using a 12-month window were created using the monthly data. Given a sequence ${\left\{\alpha_{i}\right\}}_{i=1}^{N}$, an *n*-moving average is a new sequence ${\left\{s_{i}\right\}}_{i=1}^{N-n+1}$ defined from the *α_i_* by taking the average of subsequences of *n* terms as follows [19]:

$$S_{i}=\frac{1}{n}\sum_{i}^{i+n-1}\alpha_{i}\;,$$

Where:

*N*: total number of months; *N* = 372 (31 years × 12 months/year = 372)
*i*: month; *i* = 1, 2, 3, …, 372
*α_i_*: monthly data for temperature or total precipitation or pollen count for the month *i*.
*n*: number of months being averaged; *n* = 12

The annual and monthly pollen index was skewed to the right and also exhibited a heterogeneous variability that changed proportionally with the mean count. Natural log transformation was used to stabilize the variance, normalize the series, and the probability values (p-values) for all subsequent statistical analyses of pollen concentration were based on the transformed scale. The start and end date of the birch pollen season were when the accumulated pollen concentration reached 5% and 95% of the annual total [20]. Univariate statistical methods including correlation and regression analyses and graphical techniques were also used to examine the relationship between seasonal weather data and birch pollen aerobiology including annual and daily maximum pollen concentration, the number of days from the beginning of the year to the date of maximum pollen count, as well as the starting, ending and duration of birch pollen season. The following weather data were used as predictors: monthly total precipitation or monthly mean temperature from January to May of the current year and June to December of the prior year. The trend in monthly and annual total precipitation and mean temperature over the study period was examined using simple linear regression analyses where year is the predictor variable and annual or monthly meteorological variables were the response variables. Similarly, the trend in annual and seasonal pollen index (annual pollen index, monthly pollen index, daily maximum pollen index, and the number of days from the beginning of the year to the date of maximum pollen index) over the study period was examined using simple linear regression analyses with year as the predictor variable. A Pearson correlation analysis was used to determine the association between birch pollen season variables (starting date, ending date, and duration of pollen season) and monthly mean temperature or total precipitation. Similarly, the association between birch pollen load variables (annual pollen index, daily maximum pollen index, and the number of days from the beginning of the year to the date of maximum pollen index) was examined using Pearson correlation analysis. All statistical analyses were performed using the SAS/STAT software, version 9.2 for Windows.

### 2.5. Bonferroni Correction

In statistical tests with an alpha level of 0.05, one out of every twenty tests would be expected to generate a significant result simply by chance. The 0.05 level is a criterion for significance if an *a priori* hypothesis is constructed. However, in the current study a range of *a posteriori* (or post hoc) tests were used. To account for potential spurious significant results in the analysis, Bonferroni correction was used [19]. The result of a single test would be considered significant if its associated probability is less than the Bonferroni-adjusted level. The Bonferroni-adjusted significance level is calculated as 
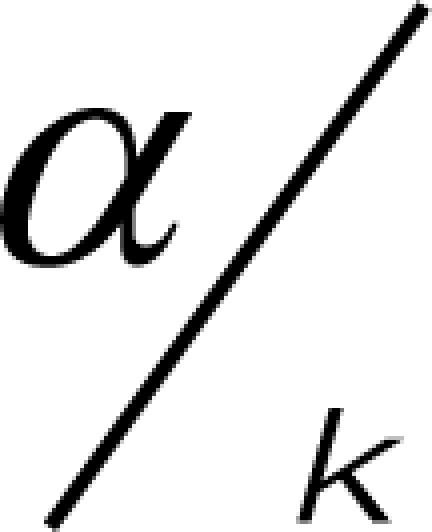
 where *α* is the standard level of significance and *k* is the number of tests. In this study, we present the Bonferroni-adjusted significance levels where it is appropriate; however, all interpretations of results were presented using the standard significance level of 0.05.

## 3. Results

### 3.1. Meteorological Data

#### 3.1.1. Long term trend in annual temperature and precipitation

**Figure 1.**
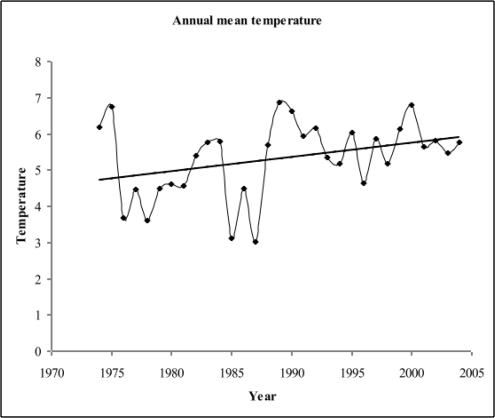
Annual mean temperature (°C) for Turku, Finland. Regression analysis was used to detect long-term trends in temperature (R^2^ = 0.123, p-value = 0.0533).

During the study period (1974–2004), the annual average temperature ranged from 3.0 °C (1987) to 6.9 °C (1989), with an average of 5.3 °C (SD = 1). There were two periods that were above and below the mean temperature; pre-1988 temperatures were below the long-term average and post-1988 temperatures were slightly above average (Figure 1). Annual mean temperature demonstrated an increasing trend but this was not statistically significant (p = 0.0533, Figure 1 and Table 1).

**Figure 2.**
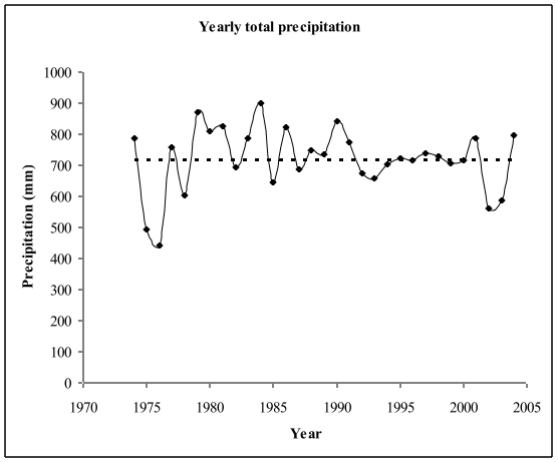
Annual precipitation (mm) for Turku, Finland. Regression analysis was used to detect long-term trends in precipitation (R^2^ = 0.00001, p-value = 0.9836).

The analysis of annual precipitation did not reveal a statistically significant change over the observed 30 years (p = 0.9836, Table 1 and Figure 2). Annual total precipitation ranged from 442.5 mm (1976) to 900 mm (1984) with a mean value of 719.9 mm (SD = 103.7).

#### 3.1.2. Long term trend in monthly temperature and precipitation

The distribution of monthly mean temperature and monthly total precipitation for the study period were examined. June, July, and August were the hottest months of the year; whereas July and August were the wettest months of the year. The study area also received a significant amount of precipitation during the fall and early winter. In Table 1, the statistical analysis demonstrated that temperature in April, July, and August changed significantly over the observed study interval. From an aerobiological perspective, emphasis should focus on identifying trends during spring since birch actively develops inflorescences in birch flowers prior to bud burst during this period [15].

### 3.2. Birch Pollen Data

#### 3.2.1. Trend in annual and seasonal pollen count

A statistically significant increase was observed in the annual accumulated pollen sum over the entire study period. The annual accumulated birch pollen sum ranged from 590 grains (1994) to 70,445 grains (1993; Table 2).

**Figure 3.**
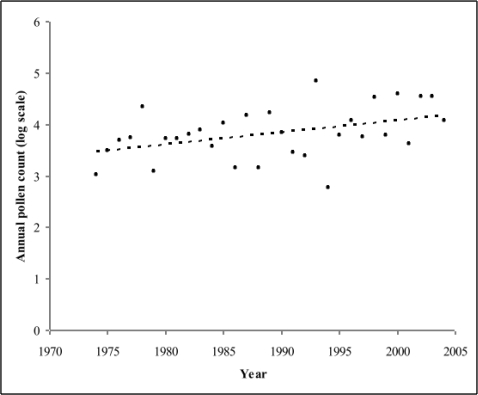
The trend in log-transformed annual cumulative birch pollen sum for the study period covering 1974–2004 and the fitted regression line (R^2^ = 0.174, p-value = 0.0197).

The variability of the annual cumulative pollen time series increased with increasing total pollen count indicating a heterogeneous variance. As a result, the annual cumulative pollen counts were log transformed to normalize the series and stabilize the variance. Regression analyses using the log transformed annual pollen count data demonstrated a significant linear increase in pollen concentrations during the study interval (R^2^ = 0.174, p = 0.0197, Table 2, Figure 3).

**Figure 4.**
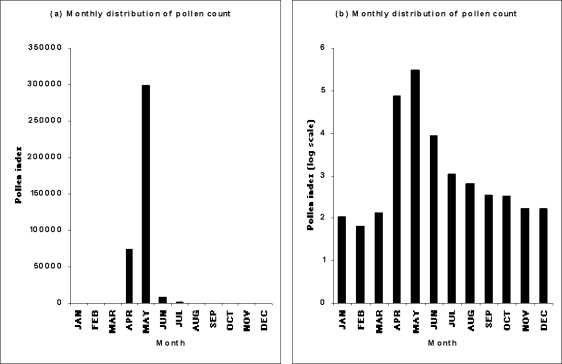
Distribution of monthly total birch pollen index for the study period (1974–2004) using actual (a) and log-transformed index (b).

The distribution of total monthly pollen counts is shown in Figure 4. April and May were the predominant months with elevated concentrations of airborne birch pollen in the atmosphere of Turku, Finland. Regression analyses demonstrated a strong and significant increase in pollen counts during April over the study period (R^2^ = 0.429, p < 0.0001; Table 3).

#### 3.2.2. Behavior of pollen release and pollen transport

The maximum daily birch pollen count and the date of occurrence were estimated for each year (Table 2). On average, maximum counts occurred on the 9^th^ day of May (number of days from the beginning of the year = 129.3, 95% CI = 125.6–133.0). The daily maximum pollen concentration (R^2^ = 0.18, p = 0.0175, Table 2) was observed to significantly increase over the study interval. In addition, the number of days before maximum daily pollen occurrence showed a trend towards earlier pollen release during later years (R^2^ = 0.287, p = 0.0019).

#### 3.2.3. Birch pollen season

**Figure 5.**
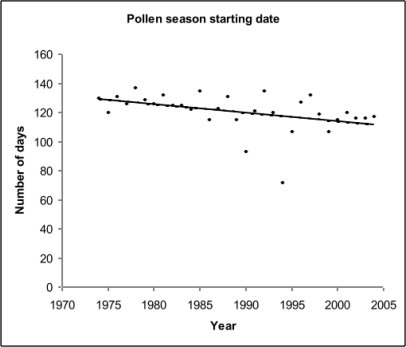
Regression analysis of the onset of the birch pollen season for the study period 1974–2004. (R^2^ = 0.161, slope −0.5762, p = 0.0252).

The start and end of the birch pollen season was estimated as the number of days from the beginning of the year (January 1^st^) to the dates when the accumulated pollen concentration reached 5% and 95% of the annual total. Using this approach, the birch pollen season commenced between April 30/May 1 and ended on May 31. The mean number of days from the beginning of the year to the start and end of the pollen season were 120.6 days (95% CI = 115.8–125.4) and 151.1 days (95% CI = 143.4–158.8), respectively. The average duration of the pollen season was 30.5 days (95% CI = 20.6–40.4). The two exceptions when the pollen season start date was premature were observed in 1990 (started early April, 93 days from the beginning of the year) and 1994 (started mid March, 72 days from the beginning of the year). The decreasing trend in the number of days to the start of the birch pollen season across the study period (R^2^ = 0.161, p = 0.0252, Figure 5) indicates a tendency for earlier pollen release (earlier season start) during later years. No significant trends were observed for the other birch parameters including the ending date and duration of the pollen season.

#### 3.2.4. Associations between birch pollen season and meteorological parameters

Pearson correlation analysis of the different aspects of the birch pollen season (beginning, end, and duration of the pollen season) with the meteorological data (temperature and precipitation of the current and prior year) is demonstrated in Table 4. Significant negative correlations were observed between the starting date of the birch pollen season and April temperature (r = −0.59, p = 0.0005) of the current year. In contrast, significant positive correlations were observed with temperature in November of the prior year (r = 0.40, p = 0.0305). These data suggest that elevated temperature in April favors earlier pollen development and release, whereas warmer temperatures recorded in November may alter the vernalisation period and favor a delayed start to the Birch pollen season. Significant negative correlations were also observed between the ending date of the pollen season and temperature in February (r = −0.36, p = 0.0448) of the current year and June (r = −0.45, p = 0.0133) and September (r = −0.63, p = 0.0002) of the prior year. Duration of the pollen season did not show any statistically significant associations with monthly temperature of the current year, however, significant negative correlations were observed with prior year temperature in June (r = −0.40, p = 0.0300) and September (r = −0.47, p = 0.0084). Statistically significant negative correlations were also observed between precipitation in January (r = −0.42, p = 0.0194) and March (r = −0.53, p = 0.0023) and the start date of the pollen season, whereas precipitation in March (r = 0.39, p = 0.0302) and April (r = 0.36, p = 0.0499) was positively correlated with duration of the pollen season. These findings suggest that temperature in the months preceding the pollen season favor the early release of pollen. The influence of other meteorological parameters such as precipitation in association with temperature will be explored in future studies using hierarchical multiple regression analysis.

#### 3.2.5. Correlation of birch pollen concentration (annual, daily maximum, and date of daily maximum) with temperature and precipitation

Pearson correlation analysis of the annual cumulative pollen sum, daily pollen count, and date of maximum daily pollen count with seasonal temperature and precipitation is shown in Table 5. Significant negative correlations were observed between the number of days from the beginning of the year to the maximum daily occurrence of birch pollen and the mean temperature in April (r = −0.61, p = 0.0002), mean temperature of February to April (FMA, r = −0.48, p = 0.0055), and mean temperature of February to May (FMAM, r = −0.46, p = 0.0084) of the current year. Statistically significant negative correlations were identified between temperature in July and September of the prior year and the number of days from the beginning of the year to the maximum daily occurrence of pollen. In contrast, significant positive correlations were observed between temperature in July and September of the prior year and the annual pollen index and the daily maximum pollen index (Table 5).

#### 3.2.6. The number of days with daily birch pollen concentration exceeding 10, 100, and 1000 grains/m^3^ of air during seasons 1974–2004

**Figure 6.**
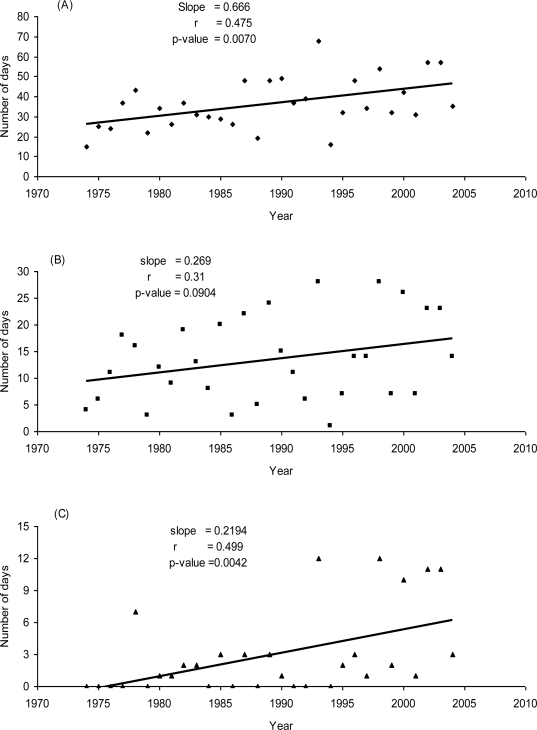
The trend in the number of days when daily average birch pollen concentration exceeded 10 (A), 100 (B), and 1000 (C) grains/m^3^ of air for each season. The slope of the regression line, the correlation coefficient and the p-value are shown.

The number of days when the daily average birch pollen load exceeded ≥ 10 grains/m^3^ of air significantly increased throughout the duration of the study (p = 0.0070; Figure 6A). This was also observed in data derived from when the daily pollen load exceeded 1000 grains/m^3^ (p = 0.0042; Figure 6C). Daily average birch pollen counts ≥100 grains/m^3^ were regularly recorded during the last 11 years, but prior to this period daily average birch pollen counts were predominantly < 100 grains/m^3^.

## 4. Discussion

The results from this retrospective study show statistically significant increases in the annual and daily average birch pollen concentrations over the 31-year study period. The timing of the birch pollen seasons were also shown to commence earlier each year throughout the duration of the study. Similar results have been previously reported in Leiden, The Netherlands [21], and Derby, England [8]. Our analyses additionally demonstrated statistically significant correlations between the onset of the birch pollen season and daily average temperature recorded in April. These results are in agreement with previous European studies that have reported associations between spring warming events and earlier starting dates of the birch pollen season [9,15,16,22–25]. Meteorological parameters in the preceding year influence aspects of the birch pollen season as demonstrated in previous investigations that have shown that the initiation and growth of male catkins, until the formation of microspores, occurs during the year before flowering [26]. The amount of birch pollen recorded in a season can also be related to meteorological variables of the preceding year [24,27]. Moreover, climate change in spring has also been proposed to affect the timing and incidence of spring flowering events [28–31]. These findings, in addition to the results presented in this study, further demonstrate that pre-seasonal temperature increases in spring could break winter dormancy and cause birch pollen seasons to commence earlier.

Alternative parameters such as long distance pollen dispersal in association with meteorological parameters have also been proposed to influence the onset of the birch pollen season in other regions of Scandinavia [11,32]. Long distance birch pollen dispersal can contribute significant concentrations of pollen to the aerospora of other geographic locations in Europe [11,32,33]. In the present study, it is possible that the earlier birch pollen season start dates observed in 1990 and 1994 resulted from the contribution of long distance birch pollen derived from other regional sources. This is an important consideration for the future interpretation of aerobiological data derived from Turku, Finland.

Trends in elevated annual birch pollen counts may also be influenced by retrospective impacts of urbanization on local *Betula* spp. communities. The supplementary planting of ornamental birch species throughout Turku and the size of residential Turku environments are variables that may lead to the spatial intensification of the airborne pollen biomass. Unlike several studies located in the United Kingdom [27,34,35] and Denmark [36], the majority of longitudinal European airborne pollen analyses have not addressed these aspects. It appears that this scenario, compared to long distance transport of pollen, could also be important predicting parameters of the birch pollen season in Turku, Finland. Furthermore, other urbanization impacts, such as soil nutrification, habitat fragmentation, disturbance, and the urban heat island effect may have enhanced the growth rates of local birch species. These aspects may directly enhance regional birch phenology. It is hypothesized that temporal increases in plant growth will result in exponential increases in inflorescence presentation. Although these aspects were not directly explored in the present study, future studies should explore these parameters when interpreting birch pollen data derived from retrospective datasets.

During the study period, statistically significant increases in the number of days where daily average birch pollen counts exceeded 10 and 1000 grains/m^3^ were observed. Increases in the duration of the birch pollen season, in addition to daily pollen concentrations greater than 10 grains/m^3^ of air, represent a significant risk factor for the exacerbation of respiratory morbidity in birch sensitized individuals [2]. Longitudinal epidemiological studies have shown decadal increases in the prevalence of allergic sensitization and asthma in developed countries [1,37–40]. It is commonly hypothesized that these increases are associated with environmental factors, personal hygiene, and improved standards of living [39,41]. However, the results of this study indicate that the higher birch pollen burden recorded during the last decade may also function as a predisposing factor for the development of atopic sensitization in Turku, Finland. Moreover, Scandinavian studies demonstrate that personal exposure to elevated amounts of birch pollen during infancy is a risk factor for the development of birch pollen allergic sensitization [42,43], and even asthma [44]. Similar observations have been made with other environmental aeroallergens, including grass, mugwort, and olive pollen [45], as well as dust mite allergens [46].

In conclusion, we have shown significant increases in the annual birch pollen index and maximum daily average birch pollen concentrations recorded at Turku during the last 31 years. The onset of birch pollen seasons was also observed to occur earlier over the study period. The onset of the birch pollen season was also associated with daily average temperature in April. In addition to these findings, the number of days where elevated concentrations exceeded 10 and 1000 grains/m^3^ air increased throughout the duration of the study. Our results suggest that elevated spring temperature in months preceding the start of the birch pollen season favor earlier inflorescence development and pollen dispersal. To date, the public health burden of elevated pollen concentrations on patients sensitized to birch have not been addressed in Turku, Finland and will be the focus of future research.

## Figures and Tables

**Table 1.** Summary of seasonal trends in temperature and precipitation covering the study period 1974–2004; results of regression analyses (independent variable = years, dependent variable = temperature or precipitation, n = 31). The slope of the regression line, R^2^ and the p-value for significance of the slope are given. Statistical significance is based on alpha levels of < 0.05 (*), < 0.01 (**), and the Bonferroni-adjusted level of significance < 0.004 (***).

	Temperature			Precipitation		
Month	Slope	Coefficient of determination (R^2^)	p-value	Slope	Coefficient of determination (R^2^)	p-value
Jan	0.0673	0.023	0.4159	0.4188	0.020	0.4429
Feb	0.0741	0.028	0.3711	0.1385	0.001	0.8713
Mar	0.0652	0.080	0.1221	0.3700	0.032	0.3379
Apr	0.0804	0.203	0.0109*	−0.0545	0.001	0.8917
May	−0.0094	0.003	0.7750	−0.0799	0.001	0.8782
Jun	0.0074	0.001	0.8403	0.6473	0.043	0.2637
Jul	0.0873	0.230	0.0063**	−0.0716	0.0002	0.9346
Aug	0.0706	0.163	0.0242*	−0.1536	0.001	0.8491
Sep	0.0401	0.053	0.2147	−0.8842	0.040	0.2790
Oct	0.0101	0.002	0.8060	0.0119	0.00001	0.9862
Nov	−0.0478	0.039	0.2872	−0.7388	0.024	0.4104
Dec	0.0381	0.013	0.5445	0.4399	0.014	0.5242
Annual^1^	0.0399	0.123	0.0533	0.0439	0.00001	0.9836

^1^Annual mean temperature or annual total precipitation

**Table 2.** The annual accumulated birch pollen sum, daily maximum pollen count, and number of days to the maximum daily occurrence of pollen and the date of occurrence, covering the period from 1974 to 2004, including slope of trend regression line, correlation coefficient, and probability value (based on log transformed values).

Year	Annual cumulative pollen (grains)	Daily maximum(grain/m^3^)	Number of days to daily maximum	Month	Day
1974	1065	231	137	5	17
1975	3119	646	133	5	13
1976	4931	798	136	5	15
1977	5574	679	140	5	20
1978	22194	5859	141	5	21
1979	1235	126	141	5	21
1980	5379	1004	129	5	8
1981	5413	1383	136	5	16
1982	6493	1683	128	5	8
1983	7956	1579	128	5	8
1984	3804	658	136	5	15
1985	10823	1933	139	5	19
1986	1454	194	118	4	28
1987	15291	3910	137	5	17
1988	1459	252	133	5	12
1989	17042	6321	117	4	27
1990	7078	1550	114	4	24
1991	2883	269	151	5	31
1992	2512	595	143	5	22
1993	70445	16291	122	5	2
1994	590	103	127	5	7
1995	6125	2208	111	4	21
1996	11924	3581	133	5	12
1997	5708	1207	135	5	15
1998	33933	5737	123	5	3
1999	6125	2208	111	4	21
2000	38713	8832	120	4	29
2001	4181	1659	121	5	1
2002	35215	5526	123	5	3
2003	35209	5526	123	5	3
2004	11897	1670	121	4	30
Slope of regression line	0.0533	0.0604	−0.0046		
Correlation coefficient	0.417	0.424	−0.536		
p-value	0.0197	0.0175	0.0019		

**Table 3.** Summary of monthly trends in airborne birch pollen counts covering the study period 1974–2004; results of regression analyses (independent variable = years, dependent variable = pollen count in log scale, n = 31). The slope of the regression line, R^2^ and the p-value for significance of the slope are given. Statistical significance is based on alpha levels of < 0.05 (*), < 0.01 (**), and the Bonferroni-adjusted level of significance < 0.004 (***).

Monthly	Slope	Coefficient of determination (R^2^)	p-value
Apr	0.1021	0.429	< 0.0001***
May	0.0121	0.035	0.3123
Jun	0.0010	0.0004	0.9196
Jul	0.0134	0.017	0.2297

**Table 4.** Correlation of the different birch pollen season variables (starting date, ending date, and duration of the pollen season) with monthly temperature and precipitation, covering the period from 1974 to 2004 (n = 31, the correlation coefficient and the probability value are given). Statistical significance is based on alpha levels of < 0.05 (*), < 0.01 (**), and the Bonferroni-adjusted level of significance < 0.003 (***).

		Temperature			Precipitation		
	Month	Starting date	Ending date	Duration	Starting date	Ending date	Duration
Prior Year	Jun	0.10	−0.45*	−0.40*	−0.14	0.23	0.25
	Jul	−0.26	−0.29	−0.06	0.12	0.02	−0.11
	Aug	0.16	−0.15	−0.15	−0.21	0.17	0.22
	Sep	0.08	−0.63***	−0.47**	0.31	0.00	0.08
	Oct	0.16	0.08	0.05	0.06	−0.19	−0.29
	Nov	0.40*	−0.04	−0.19	0.42*	−0.03	−0.24
	Dec	−0.04	−0.01	−0.05	−0.37*	−0.02	0.15
Current Year	Jan	−0.17	0.01	0.11	−0.42*	−0.14	0.17
	Feb	−0.01	−0.36*	−0.08	0.02	−0.19	−0.04
	Mar	−0.24	−0.12	0.18	−0.53**	0.35	0.39*
	Apr	−0.59***	−0.29	0.09	−0.21	0.24	0.36*
	May	0.14	−0.18	−0.26	0.04	−0.10	−0.02
	FMA^1^	−0.25	−0.36*	0.04	−0.25	0.06	0.24
	FMAM^2^	−0.19	−0.37*	−0.03	−0.22	0.02	0.22
	Annual^3^	−0.31	−0.19	0.15	−0.08	0.02	0.15

^1^Average temperature for February, March and April (FMA) or mean of total precipitation for FMA

^2^Average temperature for February, March, April and May (FMAM) or mean of total precipitation for FMAM

^3^Annual mean temperature or total annual precipitation

**Table 5.** Correlation of the different birch pollen load variables (annual count, daily maximum count, and number of days to maximum daily count) with monthly temperature and precipitation, covering the period from 1974 to 2004 (n=31, the correlation coefficient and the probability value are given). Statistical significance is based on alpha levels of < 0.05 (*), < 0.01 (**), and the Bonferroni-adjusted level of significance < 0.003 (***).

		Temperature			Precipitation		
	Month	Annual	Daily maximum	Number of days to daily maximum	Annual	Daily maximum	Number of days to daily maximum
Prior year	Jun	0.60**	0.58***	−0.11	−0.25	−0.17	−0.05
	Jul	0.40*	0.38*	−0.49**	0.15	0.10	0.21
	Aug	0.36	0.27	−0.05	−0.22	−0.16	−0.08
	Sep	0.45*	0.47**	−0.45*	0.02	−0.03	0.29
	Oct	−0.26	−0.15	0.09	0.20	0.31	−0.03
	Nov	−0.05	−0.05	0.29	0.05	0.09	0.23
	Dec	0.01	0.09	−0.07	−0.18	−0.12	−0.25
Current year	Jan	−0.07	−0.01	−0.21	−0.02	−0.09	−0.25
	Feb	0.21	0.25	−0.32	−0.17	−0.16	−0.08
	Mar	0.04	0.04	−0.33	−0.11	−0.03	−0.21
	Apr	0.20	0.24	−0.61***	−0.26	−0.22	−0.17
	May	0.22	0.18	−0.07	0.04	0.02	0.00
	FMA^1^	0.20	0.24	−0.48**	−0.27	−0.22	−0.20
	FMAM^2^	0.24	0.27	−0.46**	−0.24	−0.21	−0.19
	Annual^3^	−0.04	0.02	−0.43*	−0.33	−0.33	−0.06

^1^Average temperature for February, March and April (FMA) or mean of total precipitation for FMA

^2^Average temperature for February, March, April and May (FMAM) or mean of total precipitation for FMAM

^3^Annual mean temperature or total annual precipitation
